# Preeclampsia: Contemporary Concepts in Pathophysiology, Risk Stratification, Prevention and Monitoring

**DOI:** 10.3390/jcm15051944

**Published:** 2026-03-04

**Authors:** Piotr Witkowski, Bartosz Dzieński, Katarzyna Stefańska, Martyna Tomaszewicz, Magdalena Zabielska-Kaczorowska, Dariusz Wydra

**Affiliations:** 1 Department of Gynecology and Obstetrics, Medical University of Gdańsk, Mariana Smoluchowskiego 17, 80-214 Gdansk, Poland; piotr.witkowski97@gumed.edu.pl (P.W.); bartosz.dzienski@gumed.edu.pl (B.D.); dariusz.wydra@gumed.edu.pl (D.W.); 2 Department of Medical Immunology, Medical University of Gdańsk, Dębinki 7, 80-211 Gdansk, Poland; martyna.tomaszewicz@gumed.edu.pl; 3 Department of Physiology, Medical University of Gdańsk, Dębinki 1, 80-211 Gdansk, Poland; magdalena.zabielska-kaczorowska@gumed.edu.pl

**Keywords:** pathophysiology of preeclampsia, gestational hypertension, HELLP, angiogenic imbalance, proteinuria, sFlt-1/PIGF ratio, prevention strategies

## Abstract

Preeclampsia is a heterogeneous disorder affecting approximately 2–5% of pregnancies and remains a major cause of maternal and perinatal morbidity and mortality worldwide. Its clinical presentation ranges from mild, nearly asymptomatic forms to severe conditions progressing to eclampsia or HELLP syndrome. Despite significant advances in understanding its pathophysiology, preeclampsia continues to pose diagnostic and therapeutic challenges. In recent years, intensive research efforts have focused on developing comprehensive diagnostic criteria, identifying novel biomarkers, improving risk prediction models, and establishing effective preventive and monitoring strategies. However, expert opinions and clinical guidelines remain partially inconsistent. This review aims to summarize current global concepts regarding the epidemiology, pathophysiology, risk stratification, diagnosis, prevention, and monitoring of preeclampsia, with particular emphasis on emerging biomarkers and personalized approaches to patient care.

## 1. Introduction

Preeclampsia is a multi-organ disorder affecting 2–5% of pregnancies worldwide and is one of the leading causes of maternal morbidity and mortality during the peripartum period [1,2].

Moreover, it is a major cause of preterm birth. It is estimated that each year, worldwide, approximately 76,000 women and 500,000 children die as a result of preeclampsia and eclampsia. In high-income countries, due to early diagnosis, mortality is very low; however, in developing countries, it may reach up to 15%. Preeclampsia’s clinical presentation varies in severity, ranging from mild to life-threatening, highlighting the need for timely diagnosis and appropriate management. Modern definitions of preeclampsia extend beyond the previous criteria, which focused on the onset of hypertension with significant proteinuria after 20 weeks of gestation. Interpretation of diagnostic tests is challenging due to substantial variability in disease definitions, and several diagnostic criteria have been proposed in the literature. Consequently, multiple guidelines have been formulated by professional bodies worldwide. Currently, the internationally recognized definition is provided by the International Society for the Study of Hypertension in Pregnancy (ISSHP), which is endorsed by FIGO [1].

Although the precise pathophysiology of preeclampsia is not fully understood, it is thought to result from a combination of placental dysfunction, endothelial injury, angiogenic imbalance, and, increasingly recognized, immunological factors. Risk factors include a history of preeclampsia, chronic hypertension, diabetes, multiple gestation, advanced maternal age, and certain immunological disorders. Preeclampsia also poses significant public health challenges, contributing to maternal and neonatal morbidity and increasing healthcare costs globally.

This review aims to summarize current knowledge on the epidemiology, pathophysiology, risk assessment, and preventive strategies for preeclampsia, with particular emphasis on emerging biomarkers, the evolving role of immunological factors and prophylactic interventions.

## 2. Definition and Risk Factors

According to the ISSHP (International Society for the Study of Hypertension in Pregnancy), preeclampsia can be diagnosed even in the absence of proteinuria if other signs of maternal organ dysfunction are present. These may include edema, elevated serum creatinine, and abnormal liver function tests, as well as neurological manifestations such as headache and visual disturbances [3].

The ISSHP definition of PE was examined according to its maternal and uteroplacental component. Maternal factors include: new-onset proteinuria, renal insufficiency (serum creatinine of 90 μmol/L) in the absence of underlying renal disease, hepatic involvement (serum transaminases of >40 IU/L), thrombocytopenia (platelet count of <150,000/μL) or neurological complications such as altered mental status, blindness, stroke, clonus, severe headache, and persistent visual scotomata. Uteroplacental dysfunction factors include all the criteria above for maternal factors, with the addition of fetal death, FGR, and angiogenic imbalance, defined as serum PlGF < 5th percentile or sFlt-1/PlGF ratio > 95th percentile [4].

The newest definition of preeclampsia, presented by the ISSHP in 2021, is presented in Table 1.

According to The 2021 International Society for the Study of Hypertension in Pregnancy classification diagnosis & management recommendations for international practice [3], preeclampsia is a complex condition that remains incompletely understood, which contributes to differences among definitions proposed by various scientific societies. Table 2 presents the key differences in definitions according to the ISSHP, ACOG (American College of Obstetricians and Gynecologists), and NICE (National Institute for Health and Care Excellence).

Patients with preeclampsia should be closely monitored and managed in specialized care settings to reduce maternal and perinatal risks. Undoubtedly, accurate diagnosis, classification, and management are crucial for both the mother and the developing fetus. A more severe form of preeclampsia is HELLP syndrome (hemolysis, elevated liver enzymes, and low platelets). HELLP represents a serious complication of preeclampsia, with reported maternal mortality rates of up to 24% and perinatal mortality rates reaching 37% [5,6]. Given the often-unpredictable progression of HELLP syndrome, which can develop insidiously over days or escalate suddenly, accurate identification of risk factors may be helpful in predicting preeclampsia and its associated complications. Various maternal, obstetric, and demographic factors have been consistently associated with an increased risk of developing preeclampsia. Major clinical guidelines distinguish between factors that substantially increase the probability of preeclampsia (“high-risk”) and those that contribute to a moderate rise in risk (“moderate-risk”). High-risk factors consistently elevate the risk of preeclampsia and are usually associated with pre-existing maternal disorders or a significant obstetric history. Moderate risk factors are associated with a less pronounced but still clinically relevant increase in the likelihood of preeclampsia. This stratification aids clinical assessment and risk characterization, acknowledging that no single factor fully predicts disease onset but that combinations of risk determinants increase overall susceptibility. This categorization draws on overlapping risk profiles identified in major clinical guidelines (NICE 2019, ACOG 2019 and ISSHP 2021), which—despite minor differences in specific thresholds or additional factors—yield broadly similar groupings of high- and moderate-risk determinants for preeclampsia. Table 3 presents the classification of risk factors into high-risk and intermediate-risk categories.

In some models, certain sociodemographic characteristics, such as Black race or low socioeconomic status, are classified as moderate-risk, highlighting complex interactions between biological, social, and healthcare factors.

## 3. Pathophysiology

**Figure 1:**
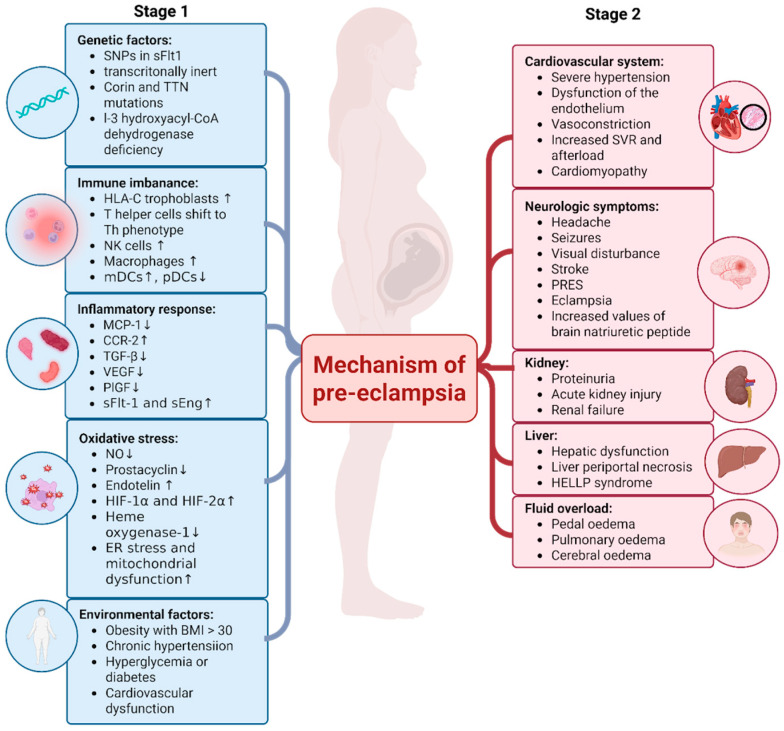
Mechanism of the development of preeclampsia in two-stage model. (↑↓) An increase or decrease in levels during pregnancy.

Despite well-established diagnostic criteria and treatment strategies, the precise etiology of preeclampsia remains incompletely understood. The pathophysiology of preeclampsia is most commonly explained by a two-stage model, in which abnormal placentation in the first stage leads to a secondary systemic maternal response. In a healthy pregnancy, fetal trophoblast cells invade and remodel the maternal spiral arteries, transforming them into dilated, low-resistance vessels. In preeclampsia, this process is incomplete, resulting in impaired uteroplacental perfusion. Subsequent placental hypoperfusion and ischemia trigger the release of pathogenic factors into the maternal circulation [7]. Moreover, hypoxia and chronic oxidative stress in the placenta lead to increased production of reactive oxygen species and concomitant reduction in antioxidant levels (such as superoxide dismutase, vitamins C and E), resulting in endothelial injury. An important role is played by the overproduction of the antiangiogenic factor soluble fms-like tyrosine kinase-1 (sFlt-1) and the reduced levels of the proangiogenic factors placental growth factor (PlGF) and vascular endothelial growth factor (VEGF). Hypoxia-induced sFlt-1 excess antagonizes VEGF and PlGF, leading to endothelial dysfunction and the development of hypertension. The subsequent section of this paper will discuss in detail the role of antiangiogenic and proangiogenic factors. Over the past few years, research on preeclampsia has increasingly focused on immunological factors. Immune system disorders associated with the development of hypertension in pregnancy may result from either an imbalance in immune cell number or dysfunction of the immune system. Recent studies show that the number of CD4 + CD25+ T regulatory cells, which normally suppress excessive inflammatory responses, is lower in the peripheral blood of women with preeclampsia [8]. Furthermore, some Treg subsets display an exhausted phenotype with a higher proportion of PD-1-positive T regulatory cells [9]. This suggests that the balance between pro- and anti-inflammatory responses is disrupted. On the other hand, researchers also suggest that PD-1 expression may be a part of a protective suppression mechanism, which in this case appears to be an insufficient response by the immune system to protect against PE induction [10]. Supporting this hypothesis, another study investigated PD-L1 interaction with decidual macrophages. Ciu et al. demonstrated that the PD-1/PDL1 axis may exert a protective effect against placental hypoxia. However, this mechanism again appears insufficient to fully prevent the onset of preeclampsia symptoms [10]. Chemokine receptor expression (e.g., CCR5, CCR4, CCR10) has also been found to be downregulated on immune cells from hypertensive women, suggesting impairment in their migratory capacity [9,11], again disrupting the immune homeostasis at the maternal–fetal interface. HIF-1α, a transcription factor activated under hypoxic conditions, promotes angiogenesis. A recent study found that treatment with an HIF-1α activator can suppress endothelial cell apoptosis and reduce oxidative stress in preeclampsia models, thereby alleviating symptoms [12,13]. However, other studies indicate that preeclamptic placentas overexpress HIF-1α, suggesting a possible role in disease induction. Further investigation is needed to clarify the precise regulatory mechanism of HIF1α activation and its role in the pathogenesis and potential treatment of PE [12]. Natural Killer (NK) cells, a key population of immune cells, play an essential role in placentation and immune tolerance. In preeclampsia, impaired uterine NK cell function reduces VEGF and PIGF secretion, leading to inadequate spiral artery remodeling and poor placenta perfusion [14]. Another contributory factor is dysregulation of the complement cascade, triggered by inflammatory activation. Placentas from PE patients exhibit increased accumulation of Membrane Attack Complexes (MACs) and decreased expression of CD55 (decay-accelerating factor, complement regulatory protein), resulting in trophoblast cell demise [15]. The full mechanism of the development of preeclampsia in the two-stage model is presented in Figure 1 [16,17,18,19,20].

## 4. Models of Preeclampsia Prediction

The ISSHP recommends that women should undergo first-trimester screening for early-onset preeclampsia using a combined test that includes maternal risk factors and biomarkers as a single-step procedure [3]. Risk assessment can be performed using the Fetal Medicine Foundation (FMF) risk calculator. 

The most effective combined screening test for preeclampsia incorporates maternal risk factors, mean arterial pressure (MAP), serum placental growth factor (PlGF) levels, and the uterine artery pulsatility index (UtA-PI) [21]. Multiple large multicenter studies have validated the effectiveness of this test, the largest of which included over 6000 pregnancies across Europe. This study reported a detection rate of 90% for very-early-onset preeclampsia, defined as preeclampsia requiring delivery before 32 weeks of gestation [22]. A high risk of preeclampsia is considered when the estimated risk equals or exceeds 1 in 100, based on the combined first-trimester screening incorporating maternal risk factors, MAP, PlGF, and UtA-PI. 

If PlGF and/or UtA-PI cannot be measured, first-trimester screening should combine maternal risk factors with MAP, rather than relying solely on maternal factors. If pregnancy-associated plasma protein A (PAPP-A) is measured as part of routine first-trimester aneuploidy screening, the result may also be incorporated into the preeclampsia risk assessment. However, studies have shown that PAPP-A is less effective in detecting preeclampsia than PlGF [23]. As presented above, the ISSHP focuses on individualized prediction models and personalized care. In contrast, organizations such as the USPSTF and NICE primarily emphasize clinical criteria and population-based prevention. As a result, there are notable differences in the frequency of preeclampsia diagnosis between the United States and Europe. The main differences in risk stratification approaches are presented in Table 4.

Undoubtedly, in the coming years, predictive criteria will continue to undergo substantial refinement and modification, driven by the rapid advances in preeclampsia diagnostics, including the development of novel biomarkers, improved imaging techniques, and increasingly sophisticated risk prediction models.

## 5. Diagnosis of Preeclampsia

According to the expanded definition of preeclampsia, all asymptomatic patients with non-severe hypertension (140–159/90–109 mmHg) and without dipstick proteinuria should undergo appropriate laboratory evaluation to rule out maternal organ dysfunction. Without such assessment, preeclampsia cannot be reliably excluded. In some cases, preeclampsia may develop in the absence of overt hypertension [24]. However, current guidelines continue to consider newly developed hypertension a key prerequisite in the diagnostic process. Below, we would like to address diagnostic methods that have evolved and undergone dynamic changes in recent years.

- **Proteinuria**

The gold standard for diagnosing proteinuria in pregnancy is a 24 h urine protein concentration, with abnormal proteinuria defined as ≥300 mg per day. In clinical practice, the 24 h urine collection is often replaced by the spot urine protein-to-creatinine ratio (uPCR), with a value of 0.3 indicating significant proteinuria [25]. The urinary protein-to-creatinine ratio (UPCR) normalizes protein excretion to the glomerular filtration rate and therefore remains unaffected by hydration status [26]. This approach overcomes the limitations associated with timed urine collections and expedites clinical decision-making. In recent years, gestational proteinuria has been recognized as a distinct clinical entity. In uncomplicated pregnancy, urinary protein excretion increases above nonpregnant baseline levels and may reach 200–260 mg/day by the third trimester in healthy women [27,28]. It has been reported that the prevalence of isolated proteinuria in pregnancy may reach 8% [25]. Increased urinary protein excretion in pregnancy is primarily attributed to the physiological increase in the glomerular filtration rate (GFR). Alterations in tubular reabsorption capacity may also contribute to this phenomenon [29,30].

- **Role of sFlt-1/PlGF ratio**

A contemporary approach to the diagnosis of preeclampsia involves the assessment of the sFlt-1/PlGF ratio. This marker is based on measuring the levels of the antiangiogenic factor soluble fms-like tyrosine kinase-1 (sFlt-1) and the proangiogenic factor placental growth factor (PlGF). In women with suspected preeclampsia, the sFlt-1/PlGF ratio demonstrates a high negative predictive value for ruling out the development of preeclampsia within the subsequent 7 days [31]. It should be emphasized that the sFlt-1/PlGF ratio is not recommended as a standalone diagnostic tool and is primarily used in combination with clinical findings and other assessments. Importantly, it serves mainly as a “rule-out” test.

The sFlt-1/PlGF ratio may be applied between 20 and 36 + 6 weeks of gestation as an adjunctive tool for short-term risk stratification and to support the establishment of a diagnosis among women at increased risk or those presenting with clinical signs suggestive of preeclampsia [32,33,34,35].

Additionally, the ratio can be utilized beyond 37 weeks of gestation in any situation where preeclampsia is suspected, or as part of ongoing assessment to quantify the extent of uteroplacental dysfunction [36,37]. 

The risk of developing preeclampsia according to the sFlt-1/PlGF ratio values is presented in Table 5 [31].

Further clinical management should be guided by the sFlt-1/PlGF ratio result. Routine monitoring of the sFlt-1/PlGF ratio is not recommended in patients without clinical features of preeclampsia. According to several studies, an exception may be made for women identified as being at high risk of developing the condition, in whom monthly assessment of the ratio could be considered. In patients presenting with features suggestive of preeclampsia, an sFlt-1/PlGF ratio below 38 effectively rules out the condition for the subsequent 2–4 weeks. Ratios between 38 and 85 warrant closer clinical surveillance and repeat testing after 1–2 weeks, or sooner if the patient’s clinical condition changes. Ratios >85 suggest that preeclampsia is likely present or imminent, necessitating intensive monitoring and management [31]. On the other hand, the PARROT-2 study demonstrated that repeat testing did not significantly reduce the rate of adverse perinatal or maternal outcomes compared with standard, single testing. Similarly, universal routine repeat testing based on PlGF in all patients with suspected preeclampsia is not recommended [38].

The sFlt-1/PlGF ratio may also be applied in twin pregnancies, offering predictive information regarding timing of delivery, risk of preeclampsia, and the development of fetal growth restriction (FGR). Threshold values differ between singleton and twin pregnancies, and the optimal cut-off point remains a subject of ongoing debate and investigation [39]. Recent theories suggest that greater placental mass, an enhanced inflammatory response, and more frequent use of assisted reproductive technologies may influence sFlt-1/PlGF levels compared to singleton pregnancies [40]. However, research groups have so far obtained inconsistent and inconclusive results. At present, the interpretation of the sFlt-1/PlGF ratio in twin pregnancies remains challenging and unclear.

## 6. Prevention Strategies

Effective prevention of preeclampsia (PE) requires an integrated, multimodal approach that combines early screening, targeted pharmacological prophylaxis, and intensive lifestyle modifications.

### 6.1. Lifestyle and Dietary Interventions

Non-pharmacological strategies focus on reducing systemic inflammation and optimizing metabolic and vascular health.

- **Physical Activity:** A 2023 systematic review and meta-analysis of randomized controlled trials found that exercise interventions during pregnancy were associated with a lower relative risk of preeclampsia (RR 0.65; 95% CI 0.42–1.03) compared with no exercise, with greater reductions observed in subgroup analyses of low-intensity or mind–body exercise modalities, although the overall effect did not reach conventional statistical significance [41].
- **Dietary Patterns:** Observational studies suggest that adherence to **healthy dietary patterns**, such as the Mediterranean or DASH diets, may reduce preeclampsia risk. The Mediterranean diet—rich in fruits, vegetables, whole grains, fish, and olive oil—was associated with **20–28% lower odds of PE** in large cohorts. Likely mechanisms include improved endothelial function, reduced inflammation, and better metabolic and blood pressure profiles. While most evidence is observational, such diets are recommended for women at higher risk of PE [42,43,44].
- Fruit and Vegetable Intake:
- Consuming at least 300–400 g of fruits and vegetables daily provides antioxidants, fiber, potassium, and polyphenols, which help modulate blood pressure and oxidative stress, further reducing PE risk [43].
- Limiting **simple sugars, saturated fats, processed meats**, and excessive sodium is important for maintaining metabolic and cardiovascular health. High intake of these components is associated with insulin resistance, systemic inflammation, elevated blood pressure, and endothelial dysfunction—all contributors to PE. Combining these restrictions with increased consumption of plant-based foods and unsaturated fats aligns with protective dietary patterns and is linked to lower incidence of preeclampsia [42].

### 6.2. Micronutrient and Vitamin Supplementation

- **Selenium:** Low-maternal-selenium status has been associated with increased oxidative stress and a higher risk of preeclampsia (PE) in observational studies. However, randomized trial evidence is limited, and routine selenium supplementation is not currently recommended solely for PE prevention [43].
- **B-Vitamins (B9 and B12):** Folate and vitamin B12 regulate homocysteine metabolism, and elevated homocysteine levels have been linked to endothelial dysfunction. Although adequate levels are essential in pregnancy, additional supplementation beyond standard prenatal recommendations has not consistently been shown to reduce PE risk [43].
- **Magnesium:** Magnesium plays a role in vascular tone regulation, but evidence supporting routine oral supplementation for PE prevention is inconclusive. Magnesium sulfate remains standard therapy for seizure prevention in severe PE, not for primary prevention [43].

### 6.3. Pharmacological Prophylaxis

- **Aspirin:**

Based on combined first-trimester screening using the Fetal Medicine Foundation Calculator, approximately 10% of pregnancies are identified as high-risk. In a predominantly Caucasian population, a risk cut-off of 1 in 100 is used to define this high-risk group [45]. Prophylactic administration of acetylsalicylic acid (aspirin) at a nightly dose of 150 mg is recommended as a standard of care. Low-dose aspirin (LDA) has been shown to be effective in reducing the risk of early-onset preeclampsia [46]. Moreover, aspirin is considered safe in pregnancy, with no evidence of increased risk for placental abruption or postpartum hemorrhage. Currently, various professional societies recommend different aspirin doses for the prevention of preeclampsia. The main differences are summarized in Table 6.

- Cost-effectiveness analysis demonstrated that universal low-dose aspirin prophylaxis is associated with a reduced incidence of preeclampsia and lower overall costs compared with no aspirin use and with aspirin administration based on serum, ultrasound, or clinical risk assessment [47].

**Table 6:** Different aspirin doses for the prevention of preeclampsia—recommendations.

Organization	Recommendation
**FMF (Fetal Medicine Foundation)**	150 mg ASA **at bedtime** from 11–14 weeks **until 36 weeks**
**ACOG (American College of Obstetricians and Gynecologists)**	81 mg ASA daily **until delivery** or at least **until 36 weeks**
**NICE (UK)**	75–150 mg ASA daily from 12 weeks **until birth**
**PTGiP (Poland)**	100–150 mg ASA at bedtime, initiated before 20 weeks, until 36 weeks
**WHO**	75 mg ASA initiated before 20 weeks of pregnancy [48]
**RANZOG**	100–150 mg daily, ideally starting before 16 weeks’ gestation and continuing until birth [48]

Ongoing studies are investigating the potential for earlier discontinuation of acetylsalicylic acid (ASA). Initial findings from large patient cohorts suggest that, in a highly specific subgroup—women with high first-trimester risk of preeclampsia and a normal sFlt-1/PlGF ratio between 24 and 28 weeks—premature cessation of ASA may be non-inferior to continuation until 36 weeks for the prevention of preterm preeclampsia [49]. Early discontinuation may also reduce the risk of minor hemorrhagic events, term-pregnancy complications, maternal anxiety, treatment costs, frequency of visits, ultrasound examinations, and iatrogenic interventions [50]. Current evidence indicates that continued aspirin therapy until 36 weeks may be largely ineffective for certain high-risk patients, potentially rendering extended treatment unnecessary. Nevertheless, most clinical guidelines continue to recommend ASA until delivery, or at least until 36 weeks, citing limited evidence to support earlier withdrawal. Further randomized controlled trials are warranted to evaluate different ASA dosages and to provide a clearer assessment of the balance between bleeding risk and therapeutic benefit associated with earlier discontinuation.

- **Low-Molecular-Weight Heparin (LMWH):**

Low-molecular-weight heparin (LMWH) may be considered in carefully selected high-risk patients, particularly those with inherited or acquired thrombophilia or a history of severe placental-mediated complications. Although some data suggest a potential benefit when combined with low-dose aspirin, current evidence does not support routine LMWH administration solely for preeclampsia prevention in the absence of established thrombotic indications [44].

### 6.4. Emerging Therapies

Several small studies have investigated metformin and pravastatin as potential strategies for preeclampsia prevention.

Metformin, particularly in women with insulin resistance, obesity, or PCOS, may improve endothelial function and modulate placental angiogenic balance by reducing the release of antiangiogenic factors such as sFlt-1 [44].

Pravastatin has been explored for its pleiotropic vascular effects, including induction of the heme-oxygenase-1 (HO-1) pathway, which may attenuate oxidative stress and decrease sFlt-1 levels [44].

Despite promising mechanistic data, current clinical evidence remains limited and insufficient to support routine use of these agents. Larger, adequately powered randomized controlled trials are required to determine their efficacy and safety in preeclampsia prevention.

Prevention of preeclampsia remains a critical component of maternal healthcare. Among the various interventions studied, aspirin has demonstrated efficacy in high-risk populations. Novel therapies, including metformin and L-arginine, warrant further investigation. Despite advances in prophylaxis and clinical care for high-risk pregnancies, the development of new preventive strategies remains a priority to further reduce the maternal and neonatal complications associated with preeclampsia.

## 7. Monitoring and Surveillance of Women with Preeclampsia

If preventive measures are insufficient and a patient develops hypertension complicated by preeclampsia, close monitoring becomes essential. In this context, management strategies differ across countries and healthcare systems worldwide. 

The ISSHP guidelines are highly individualized, similarly to their diagnostic criteria. In addition to regular blood pressure measurements and clinical assessment (headache, visual disturbances, and epigastric pain), the ISSHP recommends monitoring laboratory parameters, with particular attention on platelet count, serum creatinine, and liver function tests. Fetal well-being must also be regularly assessed through ultrasound evaluation of fetal growth, Doppler studies, and cardiotocography (CTG). In selected cases, the sFlt-1/PlGF ratio may be measured.

In contrast, ACOG stratifies patients into high- and low-risk groups. For women without severe features of preeclampsia, blood pressure should be monitored at least twice weekly, and laboratory tests, CTG, and ultrasound should be performed every 1–2 weeks. These patients can be managed on an outpatient basis. In cases of preeclampsia with severe features, hospitalization is recommended, and maternal and fetal parameters should be monitored daily. ACOG does not recommend the routine use of biomarkers for monitoring.

NICE proposes a structured surveillance approach, including regular blood pressure measurements (daily during hospitalization or at least twice weekly in outpatient care), monitoring of proteinuria, laboratory tests performed 1–2 times per week, and fetal ultrasound every 2–4 weeks. NICE also allows the use of PlGF/sFlt-1 testing in differential diagnosis.

## 8. Timing and Mode of Delivery in Women with Established Preeclampsia

International guidelines consistently emphasize that delivery is the definitive treatment for preeclampsia. According to the ISSHP, ACOG, and NICE, women with preeclampsia without severe features should generally be delivered at 37 weeks of gestation. In cases with severe features, delivery is recommended at or beyond 34 weeks, or earlier in the presence of maternal or fetal deterioration. Before 34 weeks of gestation, expectant management may be considered in carefully selected and clinically stable patients managed in tertiary care centers. The indications for immediate delivery are presented in Table 7. 

These recommendations highlight the need to balance maternal safety with fetal maturity and to individualize clinical decision-making.

## 9. Conclusions

Preeclampsia is a heterogeneous disorder, and knowledge about it is evolving dynamically. We are witnessing significant changes in its definition, preventive strategies, risk stratification, and the search for new diagnostic methods in order to appropriately monitor and treat affected patients. The complexity of this condition is reflected, among other factors, in the wide variation in approaches to its definition worldwide. Undoubtedly, an individualized approach to each patient is essential.

## Figures and Tables

**Table 1:** Criteria for preeclampsia according to ISSHP (2021).

Preeclampsia (de novo) is gestational hypertension accompanied by one or more of the following new-onset conditions at ≥20 weeks’ gestation: Proteinuria Other maternal end-organ dysfunction, including: Neurological complications (e.g., eclampsia, altered mental status, blindness, stroke, clonus, severe headaches, or persistent visual scotomata); Pulmonary edema; Hematological complications (e.g., platelet count <150,000/μL, DIC, hemolysis); Acute Kidney Injury—AKI (such as creatinine ≥90 μmol/L or 1 mg/dL); Liver involvement (e.g., elevated transaminases such as ALT or AST >40 IU/L) with or without right upper quadrant or epigastric abdominal pain). Uteroplacental dysfunction (e.g., placental abruption, angiogenic imbalance, fetal growth restriction, abnormal umbilical artery Doppler waveform analysis, or intrauterine fetal death).

**Table 2:** Key differences in definitions of preecklampsia according to ISSHP, ACOG, and NICE.

Domain	ISSHP	ACOG	NICE
**Proteinuria**	Not required	Not required	Still emphasized
**Placental dysfunction**	Included	Not included	Partially considered
**Biomarkers** **(PlGF, sFlt-1)**	Supported	Not Recommended	Selective use
**Scope**	Broadest	Narrowest	Intermediate

PIGF = placental growth factor; sFlt-1 = soluble fms-like tyrosine kinase-1.

**Table 3:** Classification of risk factors.

High-Risk Factors	Moderate-Risk Factors
Prior history of preeclampsia, chronic hypertension, pre-gestational diabetes mellitus, chronic kidney disease, systemic lupus erythematosus, antiphospholipid syndrome, multifetal pregnancy.	Nulliparity, advanced maternal age (commonly defined as ≥35 years in ACOG and ≥40 years in some classifications), maternal obesity (e.g., body mass index thresholds ≥30 kg/m^2^ or ≥35 kg/m^2^), long interpregnancy interval (e.g., >10 years).

**Table 4:** Main differences in risk stratification approaches.

Criterion	USPSTF (USA)	NICE (UK)	ACOG (USA)	ISSHP (International)
**Primary approach**	Risk factor-based prevention	Risk factor-based prevention	Prevention and diagnostic support	Prevention and advanced diagnostics
**Eligibility criteria**	≥1 high-risk or ≥2 moderate-risk factors	≥1 high-risk or ≥2 moderate-risk factors	≥1 high-risk or ≥2 moderate-risk factors	Individualized risk assessment
**First-trimester prediction models**	Not recommended	Limited application	Limited application	Recommended
**MAP**	Not recommended	Not recommended	Not recommended	Recommended
**UtA-PI**	Not recommended	Not recommended	Not recommended	Recommended
**Personalized medicine**	Low	Low	Moderate	High

MAP = mean arterial pressure; UtA-PI = uterine artery pulsatility index.

**Table 5:** The risk of developing preeclampsia according to the sFlt-1/PlGF ratio values.

Low Risk	Moderate Risk	High Risk
<38	38–85	>85

**Table 7:** The indications for immediate delivery.

The Indications for Immediate Delivery
Eclampsia, HELLP syndrome, pulmonary edema, uncontrolled severe hypertension, non-reassuring fetal status.

## Data Availability

No new data was created or analyzed in this study.
